# Editorial: Beef on Dairy: The Use of a Simple Tool to Improve Both Cattle Production Systems

**DOI:** 10.3389/fgene.2022.813949

**Published:** 2022-04-26

**Authors:** Rodrigo R. Mota, Luiz F. Brito, Donagh P. Berry

**Affiliations:** ^1^ Council on Dairy Cattle Breeding (CDCB), Bowie, MD, United States; ^2^ Department of Animal Sciences, Purdue University, West Lafayette, IN, United States; ^3^ Teagasc, Animal & Grassland Research and Innovation Centre, Moorepark, Ireland

**Keywords:** crossbreeding, beef-on-dairy, genomic prediction, GWAS, reproductive technologies

Crossbreeding schemes have been widely used in global beef and dairy cattle (Pahmeyer and Britz, 2020; Quénon et al., 2020). This breeding strategy has been historically used, especially in tropical regions, to develop more productive and climatically-adapted populations such as the Girolando (Costa et al., 2020) and Montana Tropical Composite (Grigoletto et al., 2020) populations. The term “Beef-on-dairy” is growing in popularity and is simply the mating of beef bulls to dairy females, with the resulting progeny generally used for meat production (Berry, 2021). Interest in this mating type is increasing for a multitude of reasons including: 1) a reducing requirement for dairy-on-dairy matings owing to both improving reproductive performance in many dairy cow populations coupled with the greater availability of sexed semen from more genetically elite dairy sires without incurring a large reduction in conception rate, 2) a desire to maintain cash flow through the sale of surplus progeny for meat production thus providing resilience to volatile milk price, and 3) growing consumer interest in how food is produced and how it aligns with their social values; the often close to zero value of male dairy-bred calves is of concern to many. Crosses between dairy and beef animals are expected to give maximum heterosis given the difference in breed origins. Despite the growing use of beef-on-dairy matings, many questions still remain across the entire beef-on-dairy production system all the way from conception to marketing. Therefore, the main motivation for this special topic was to compile state-of-the-art research investigating management practices, economic assessments, and breeding tools for improving dairy-beef production systems. Of particular interest is the role of modern technologies in delivering on the goals of efficient beef-on-dairy production systems.

This special Research Topic includes six papers presenting novel and exciting breeding strategies and methods such as genomic prediction of breeding values, genome-wide association studies, beef-cross-dairy performance, and reproductive technologies. Fitting haplotypes instead of single nucleotide polymorphism (SNP) markers in genomic prediction models was investigated as an approach to improve the accuracy of genomic breeding values (Feitosa et al., 2020; Teissier et al., 2020; Araujo et al., 2021). In this context, Li et al. evaluated the predictive performance of carcass traits in Chinese Simmental cattle using haplotypes in comparison to the traditional genomic prediction approaches. In comparison to the individual SNP-based genomic prediction methods, the authors reported that using haplotypes resulted in more accurate genomic breeding values for carcass traits in Chinese Simmental cattle. Furthermore, the authors indicated that a haplotype strategy considering biological information can also be used to identify variants affecting complex traits of economic importance.

Verification that genetic evaluations materialize as phenotypic differences is fundamental to stakeholder acceptance and uptake. Martín et al. evaluated beef-x-dairy offspring performance based on their sire predicted breeding values and concluded that estimated breeding values derived from beef-breed data can be useful, albeit the response to selection in beef-on-dairy progeny was half that in beef-on-beef progeny. The study concluded that New Zealand farmers should consider the use of sire estimated breeding values for improved offspring performance in terms of beef production since the performance of beef-cross-dairy offspring was a function of their sire’s EBV. In addition to sire selection for beef-on-dairy matings, Berry and Ring advised how understanding dairy farmer attitude to sire selection depending on the market outlet for the resulting surplus calves can provide valuable information for the animal breeding chain. Using a large dataset from Irish commercial dairy herds, Berry and Ring concluded that dairy producers who rear their surplus progeny post weaning, on average, chose dairy and beef sires with superior expected genetic merit for carcass traits.

According to Li and Cabrera (2019), beef price will increase up to 2028 due to the growing population, and consequently, higher demand. Therefore, the authors pointed out that measures should be taken to meet this demand, such as the optimal use of beef semen in dairy herds. Thus, the use of reproductive technologies is an essential part to improve productive efficiency. However, reproductive technologies are not always affordable in certain regions of the world or production systems, especially for small-holder farmers in developing countries. In this regard, Nengovhela et al. evaluated the impact of using reproductive technologies to improve productivity in low-income beef farms. Their study revealed that many factors may affect the adoption of reproductive technologies by the low-income beef sector but its implementation is still recommended because of the high success rate and better reproductive performance observed where this technology was implemented.

Milk fatty acids and milk protein are key traits in the dairy industry. Khan et al. conducted an interesting review on the association of the *DGAT1* gene with milk and meat production traits in cattle, buffalo, goats, and sheep. These authors demonstrated that *DGTA1* has a positive influence on meat and carcass fatness in beef cattle along with meat production in sheep and goats; *DGTA1* also regulates milk production in sheep, goats, and buffaloes. On the other hand, Cai et al. studied the miRNA profiles of mammary glands from twelve Chinese Holstein cows and provided new insights into the mechanism of milk protein synthesis.

Although the six articles published in this section encompasses broad subjects, gaps in knowledge still exist. For instance, knowledge gaps exist in the use of sexed semen and *in vitro* production (IVP) in beef-on-dairy systems, environmental credentials of dairy-beef vs. beef systems, development of new genomic evaluation systems for crossbred performance, genotype-by-environment (GxE) interactions in different production systems, the impact of non-additive genetic effects on the phenotypic variability of relevant traits in crossbred populations, and breeding strategies considering the combining ability of beef and dairy animals with particular reference to 1) exploiting location-specific heterosis across the genome through mating advice algorithms, and 2) achieving a compromise in traits such as calving difficulty from beef-on-dairy matings. We hope that this special research topic will motivate future studies aiming to maximize the success of beef-on-dairy production system.

## References

[B1] Araujo A. C. Carneiro P. L. S. Oliveira H. R. Schenkel F. S. Veroneze R. Lourenco D. A. L. (2021). A Comprehensive Comparison of Haplotype-Based Single-Step Genomic Predictions in Livestock Populations with Different Genetic Diversity Levels: A Simulation Study. Front. Genet. 12, 729867. 10.3389/fgene.2021.729867 34721524 PMC8551834

[B2] Berry D. P. (2021). Invited Review: Beef-On-Dairy-The Generation of Crossbred Beef × Dairy Cattle. J. Dairy Sci. 104 (4), 3789–3819. 10.3168/jds.2020-19519 33663845

[B3] Costa N. S. Silva M. V. G. Panetto J. C. D. C. Machado M. A. Seixas L. Peripolli V. (2020). Spatial Dynamics of the Girolando Breed in Brazil: Analysis of Genetic Integration and Environmental Factors. Trop. Anim. Health Prod. 52 (6), 3869–3883. 10.1007/s11250-020-02426-z 33094421

[B4] Feitosa F. L. B. Pereira A. S. C. Amorim S. T. Peripolli E. Silva R. M. d. O. Braz C. U. (2020). Comparison between Haplotype‐Based and Individual Snp‐Based Genomic Predictions for Beef Fatty Acid Profile in Nelore Cattle. J. Anim. Breed. Genet. 137 (5), 468–476. 10.1111/jbg.12463 31867831

[B5] Grigoletto L. Ferraz J. B. S. Oliveira H. R. Eler J. P. Bussiman F. O. Abreu Silva B. C. (2020). Genetic Architecture of Carcass and Meat Quality Traits in Montana Tropical Composite Beef Cattle. Front. Genet. 11, 123. 10.3389/fgene.2020.00123 32180796 PMC7057717

[B6] Li W. Cabrera V. E. (2019). Dairy × Beef : Fad or Sustainable Future ? Proceedings JSS. Available at: https://dairymgt.info/publications/common_files/Cabrera Proceedings_JSS_CLEAN COPY 0921.pdf (October 30, 2021).

[B7] Pahmeyer C. Britz W. (2020). Economic Opportunities of Using Crossbreeding and Sexing in Holstein Dairy Herds. J. Dairy Sci. 103 (9), 8218–8230. 10.3168/jds.2019-17354 32684478

[B8] Quénon J. Ingrand S. Magne M.-A. (2020). Managing the Transition from Purebred to Rotational Crossbred Dairy Cattle Herds: Three Technical Pathways from a Retrospective Case-Study Analysis. Animal 14 (6), 1293–1303. 10.1017/S1751731119003458 31959276

[B9] Teissier M. Larroque H. Brito L. F. Rupp R. Schenkel F. S. Robert-Granié C. (2020). Genomic Predictions Based on Haplotypes Fitted as Pseudo-SNP for Milk Production and Udder Type Traits and SCS in French Dairy Goats. J. Dairy Sci. 103 (12), 11559–11573. 10.3168/jds.2020-18662 33041034

